# H3K56me3 Is a Novel, Conserved Heterochromatic Mark That Largely but Not Completely Overlaps with H3K9me3 in Both Regulation and Localization

**DOI:** 10.1371/journal.pone.0051765

**Published:** 2013-02-22

**Authors:** Antonia P. M. Jack, Silva Bussemer, Matthias Hahn, Sebastian Pünzeler, Martha Snyder, Michael Wells, Gyorgyi Csankovszki, Irina Solovei, Gunnar Schotta, Sandra B. Hake

**Affiliations:** 1 Center for Integrated Protein Science Munich (CIPSM) at the Adolf-Butenandt-Institute, Department of Molecular Biology, Ludwig-Maximilians-University Munich, Munich, Germany; 2 Department of MCDB, University of Michigan, Ann Arbor, Michigan, United States of America; 3 LMU Biozentrum, Department of Biology II, Ludwig-Maximilians-University Munich, Planegg-Martinsried, Germany; St Jude Children's Research Hospital, United States of America

## Abstract

Histone lysine (K) methylation has been shown to play a fundamental role in modulating chromatin architecture and regulation of gene expression. Here we report on the identification of histone H3K56, located at the pivotal, nucleosome DNA entry/exit point, as a novel methylation site that is evolutionary conserved. We identify trimethylation of H3K56 (H3K56me3) as a modification that is present during all cell cycle phases, with the exception of S-phase, where it is underrepresented on chromatin. H3K56me3 is a novel heterochromatin mark, since it is enriched at pericentromeres but not telomeres and is thereby similar, but not identical, to the localization of H3K9me3 and H4K20me3. Possibly due to H3 sequence similarities, Suv39h enzymes, responsible for trimethylation of H3K9, also affect methylation of H3K56. Similarly, we demonstrate that trimethylation of H3K56 is removed by members of the JMJD2 family of demethylases that also target H3K9me3. Furthermore, we identify and characterize mouse mJmjd2E and its human homolog hKDM4L as novel, functionally active enzymes that catalyze the removal of two methyl groups from trimethylated H3K9 and K56. H3K56me3 is also found in *C. elegans*, where it co-localizes with H3K9me3 in most, but not all, tissues. Taken together, our findings raise interesting questions regarding how methylation of H3K9 and H3K56 is regulated in different organisms and their functional roles in heterochromatin formation and/or maintenance.

## Introduction

Histones, the building blocks of chromatin, are subject to several posttranslational modifications including methylation, acetylation and phosphorylation that carry important functional information [1]. Over the last decades, it has become increasingly obvious that such chemical histone tags contribute to the regulation of DNA-related processes in a highly selective and specialized manner [2]. These posttranslational histone modifications (PTMs) either change nucleosome structure directly by affecting histone-DNA contacts or indirectly by recruiting PTM-binding proteins that act on the underlying chromatin structure, as has been proposed in the “histone code” hypothesis [3]. Although most marks are found on the flexible histone tail regions, some modifications have also been identified on core residues. One such core PTM, histone H3 lysine 56 acetylation (H3K56ac) [4], occurs in the α-N-helical region near the entry-exit sites of the DNA superhelix and is conserved from yeast to man [5]. It is most abundant during S phase [6], [7] and has been shown to play a pivotal role in DNA damage response [6], chromatin integrity [8], [9] and replication-coupled nucleosome assembly [10]. In a previous mass spectrometry-based study, we were not only able to verify the existence of H3K56 acetylation in humans but were also able to identify low levels of mono- and trimethylation of lysine 56 on histone H3 (H3K56me1 and H3K56me3, respectively) [11]. Recently, it was demonstrated that monomethylation of H3K56 regulates DNA replication through interaction with the replication processivity factor PCNA and is catalyzed by the lysine methyltransferase (KMT) G9a (KMT1C) [12]. The involvement of H3K56me1 in such an important biological event led us to ask how trimethylation of this residue might be regulated and impact cellular processes. Despite the known *in vivo* existence of H3K56me3 [11], no further information concerning this novel histone H3 core modification has been established. We set out to learn more about its functional role by deciphering its chromatin localization and by identifying enzymes that set (“writer”) and erase (“eraser”) this mark.

## Materials and Methods

### Cell lines

Human HeLa Kyoto cells [13], and mouse C127 (ATCC CRL-1616) cell lines were grown in DMEM medium (PAA) supplemented with 10% FCS (Sigma) and 1% penicillin/streptomycin at 37°C and 5% CO_2_. Wild type, Suv39hDKO [14] and SUV4-20hDKO [15] mouse embryonic fibroblast (MEF) cell lines were grown in DMEM medium (PAA) supplemented with 18% FCS (Sigma), 1% penicillin/streptomycin, 1% non-essential amino acids (Invitrogen), 50 mM β-mercaptoethanol and 0.4% LIF at 37°C and 5% CO_2_. Cells were transfected using FuGene HD (Roche Applied Science) according to the manufacturer's instructions.

### Antibodies

Polyclonal rabbit antibody against H3K56me3 was developed by Pineda Antikörper-Service (Berlin, Germany) using a peptide with the following amino acid sequence for immunization and affinity purification: NH_2_-CRRYQ-K(me_3_)-STEL-CONH_2_. Commercially available antibodies used in this study include: Primary antibodies: αH3 (C-terminus, Abcam), αH4 (Antikoerper-online), αH3K4me2 (Abcam), αH3K4me3 (Abcam), αH3K9me1 (Millipore), αH3K9me2 (Active Motif), αH3K9me3 (Active Motif and [16]; specificity tests are shown in Figure S1), αH3K27me2 (Millipore), αH3K27me3 (Millipore), αH3K36me1 (Millipore), αH3K36me2 (Active Motif), αH3K36me3 (Abcam), αH4K20me1 (Millipore), αH4K20me2 (Millipore), αH4K20me3 (Abcam), αH3K56me1 (Millipore), αH3K56me2 (Active Motif), αH3K56ac (Active Motif). Secondary antibodies: for immunoblots (Amersham), for IF microscopy (Dianova).

### Peptide competition experiment

αH3K56me3 antibody in 1∶1000 or 1∶100 dilutions was preincubated with 2 µg/ml of peptides (Table S1) before usage in either immunoblots or immunofluorescence (IF) microscopy, respectively. Peptides were N-terminally biotinylated and synthesized with higher than 80% purity by The Rockefeller University, GeneScript or the MPI for Biochemistry Munich. In case of immunoblots, acid extracted histones [17] and recombinant histone H3 [18] were used.

### Tryptic digest of mononucleosomes

6×10^7^ HeLa Kyoto cells were incubated in PBS, 0.3% Triton X-100 and Protease Inhibitor Cocktail (Roche, Germany) for 10 min at 4°C. Nuclei were pelleted, washed once in PBS, resuspended in EX100 buffer (10 mM Hepes pH 7.6, 100 mM NaCl, 1.5 mM MgCl_2_, 0.5 mM EGTA, 10% (v/v) glycerol, 10 mM β-glycerol phosphate, 1 mM DTT, Protease Inhibitor Cocktail (Roche, Germany)) and CaCl_2_ concentration adjusted to 2 mM. Resuspended nuclei were digested with 1.5 U MNase (Sigma) for 20 min at 26°C. The reaction was stopped by addition of EGTA to a final concentration of 10 mM followed by centrifugation for 10 min at 1000 rcf, 4°C. Mononucleosome containing supernatant was retained. NH_4_HCO_3_ was added at a final concentration of 50 mM or until a pH of 7–8 was reached.1.6 µg Trypsin (Promega) was added and the reaction was incubated at 25°C. Samples were collected at different time points and the reaction stopped by adding an equal volume of 1% trifluoroacetic acid. Fragments were size separated on a 15% SDS-PAGE probed with indicated antibodies.

### Spot-blot

Peptide dilutions containing 2, 10, 50, 200 and 1000 ng in sterile water were spotted on nitrocellulose membrane and allowed to air-dry. The membrane was then blocked in PBS-Tween (0.1%) with milk powder (5%), followed by immunoblotting with αH3K56me3.

### Immunofluorescence (IF) microscopy and cell cycle analysis

#### Mammalian cells

Preparation of mammalian cells and chromosome spreads for IF microscopy was done as previously reported [19]. Staining of S-phase cells was performed as described in [18]. Wide-field IF imaging of EdU-stained C127 cells was performed on a PersonalDV microscope system (Applied Precision) equipped with a 60×/1.42 PlanApo oil objective (Olympus), CoolSNAP ES2 interline CCD camera (Photometrics), Xenon illumination and appropriate filtersets. Iterative 3D deconvolution of image z-stacks was performed with the SoftWoRx 3.7 imaging software package (Applied Precision).

Confocal imaging of chromosome spreads was performed on a TCS SP5 II microscope system (Leica Microsystems, Wetzlar, Germany), equipped with a 63×/1.3 HCX PL APO glycerol immersion objective. Z-stacks were recorded and subsequently deconvolved with Huygens Essential Software (SVI, Hilversum, The Netherlands).

Image stacks of immunostained MEF cells were collected using a Leica TCS SP5 confocal microscope with Plan Apo Lambda Blue 63×/1.4 NA oil or 63×/1.3 glycerol immersion objective.

#### *C. elegans*

Methanol/acetone fixation for immunostaining was performed as follows. Adult hermaphrodites were dissected in 1x sperm salts with and frozen on dry-ice for 20–30 minutes. The slides were fixed in methanol followed by acetone, 2 minutes each wash, at −20°C. Slides were then washed once for ten minutes in PBST prior to incubation with primary antibody [1∶200 or 1∶100 (direct labeling) αH3K56me3, 1∶1000 αH3K9me3 (Abcam ab8898)]. Remainder of staining protocol was conducted as described previously [20]. Microscopy and imaging were conducted as described previously [21].

Images were capture with a Hamamatsu Orca-Erga close-coupled-device (CCD) camera mounted on an Olympus BX61 motorized Z-drive microscope using a 60X APO oil immersion objective. These images are projections of optical sections with a Z spacing of 0.2 micrometers. Scale bars were added using ImageJ (available at http://rsb.info.nih.gov/ij; developed by Wayne Rasband, National Institutes of Health, Bethesda, MD) and a template image created in Slidebook.

### Quantitative PCR

qPCR was carried out as previously described [22] using Fast SYBR Green Master Mix (Applied Biolabs). Results were normalized to HPRT1 and GAPDH levels.

### Cloning of GFP-jmjC constructs

pDONR entry clones of the Jmjd2 subgroup [23] were recombined into the target vector pEGFP-N1-GW using LR clonase II enzyme mix (Invitrogen) according to the manufacturer's protocol.

### *C. elegans* RNAi

RNA interference by feeding was performed with the Ahringer laboratory RNAi feeding library [24] in two generations as described previously [21].

## Results

### Development of a specific αH3K56me3 antibody

**Figure 1:**
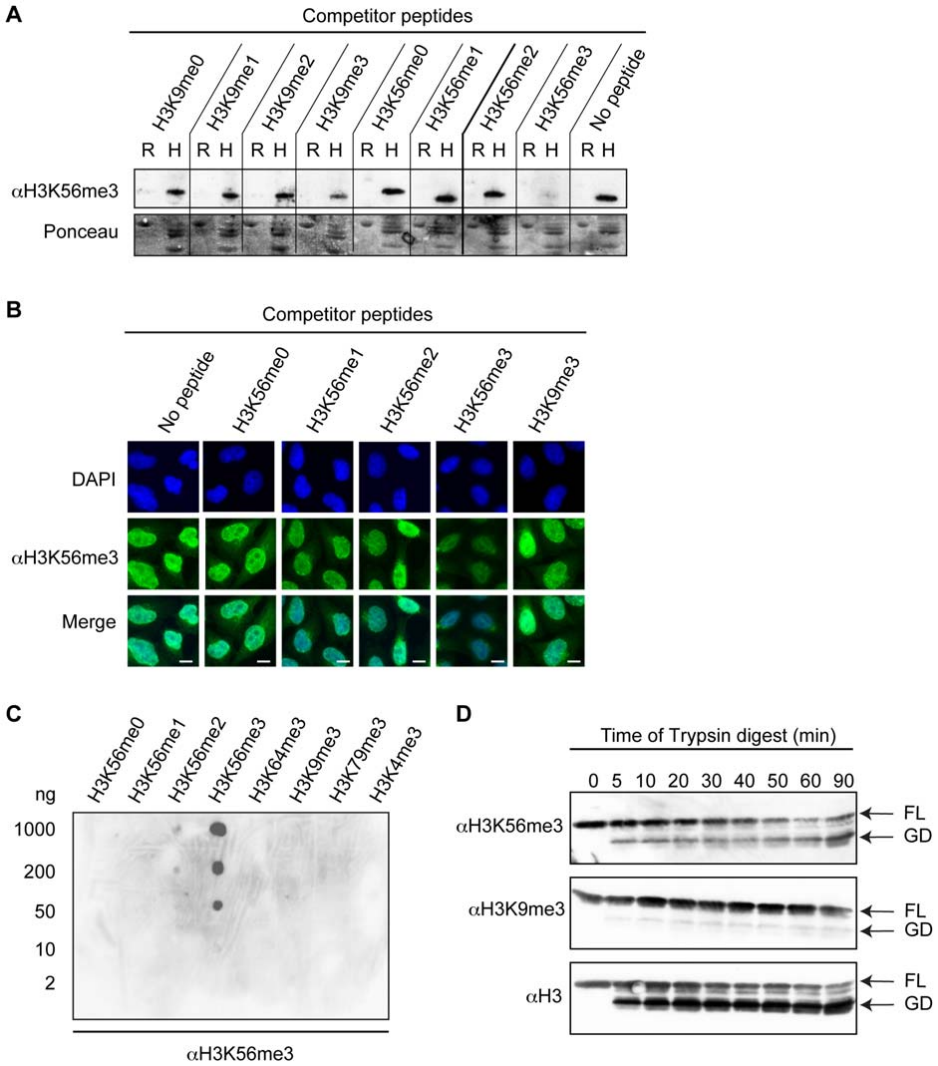
Determination of αH3K56me3 specificity and suitability in diverse applications. (A) Immunoblot peptide competition experiment. αH3K56me3 antibody was preincubated with competitor peptides before addition to immunoblots containing recombinant H3 protein (R) or acid extracted HeLa Kyoto histones (H) (top). Ponceau staining (bottom) serves as loading control. (B) IF microscopy peptide competition experiment. αH3K56me3 antibody (green) was preincubated with competitor peptides before addition to fixed HeLa Kyoto cells. DAPI (blue) stains DNA. Scale bar  = 5 µm. (C) Spot-blot with different concentrations (5–1000 ng) of H3 peptides to determine αH3K56me3-binding affinities. (D) Immunoblot of sequential tryptic digest of HeLa Kyoto-derived mononucleosomes using αH3K56me3 (top), αH3K9me3 (middle) and αH3 (bottom). FL  =  full-length histone H3, GD  =  N-terminally deleted globular domain of histone H3.

To gain insight into the biological function(s) of H3K56 trimethylation, we raised a polyclonal antibody against H3K56me3 (αH3K56me3) and determined its specificity in various assays. Since H3K56me1 has previously been reported to be catalyzed by the H3K9me1-specific KMT G9a, maybe due to a conserved lysine-serine-threonine (K/S/T) motif at the site of both residues [12], we put special emphasis on testing a potential cross-reactivity of this antibody with H3K9me3. First, we performed peptide competition experiments using peptides spanning diverse regions of histone H3 with or without different methylation states. Specific antibody recognition of H3K56me3 in immunoblotting (Figure 1A) and immunofluorescence (IF) microscopy (Figure 1B) was efficiently competed out only with H3K56me3-containing peptides, but not with peptides containing other methylated or unmethylated histone regions. Next, we determined the relative binding affinity of αH3K56me3 to its epitope by a peptide Spot-blot containing various concentrations of different histone peptides and observed that αH3K56me3 detected as low as 50 ng of H3K56me3 peptides (Figure 1C). Notably, αH3K56me3 does not recognize any other trimethylated peptides except H3K56me3. For further support of antibody specificity, we generated mononucleosomes from HeLa cells that were subsequently digested with different concentrations of Trypsin in order to generate histones lacking their flexible tail regions. In this way, we were able to determine if the antibody epitope resides in the H3 core region or N-terminal tail. In immunoblots, αH3K56me3, but not the control αH3K9me3 antibody, recognized both full-length (FL) and the N-terminus deleted globular domain (GD) of histone H3 (Figure 1D), demonstrating that αH3K56me3 specifically binds to a modification in the core region of H3. In summary, these experiments provide compelling evidence that αH3K56me3 is highly specific for this particular modification and can be applied in diverse biochemical assays.

### H3K56me3 is evolutionary conserved and localizes to pericentromeric heterochromatin outside of S-phase

**Figure 2:**
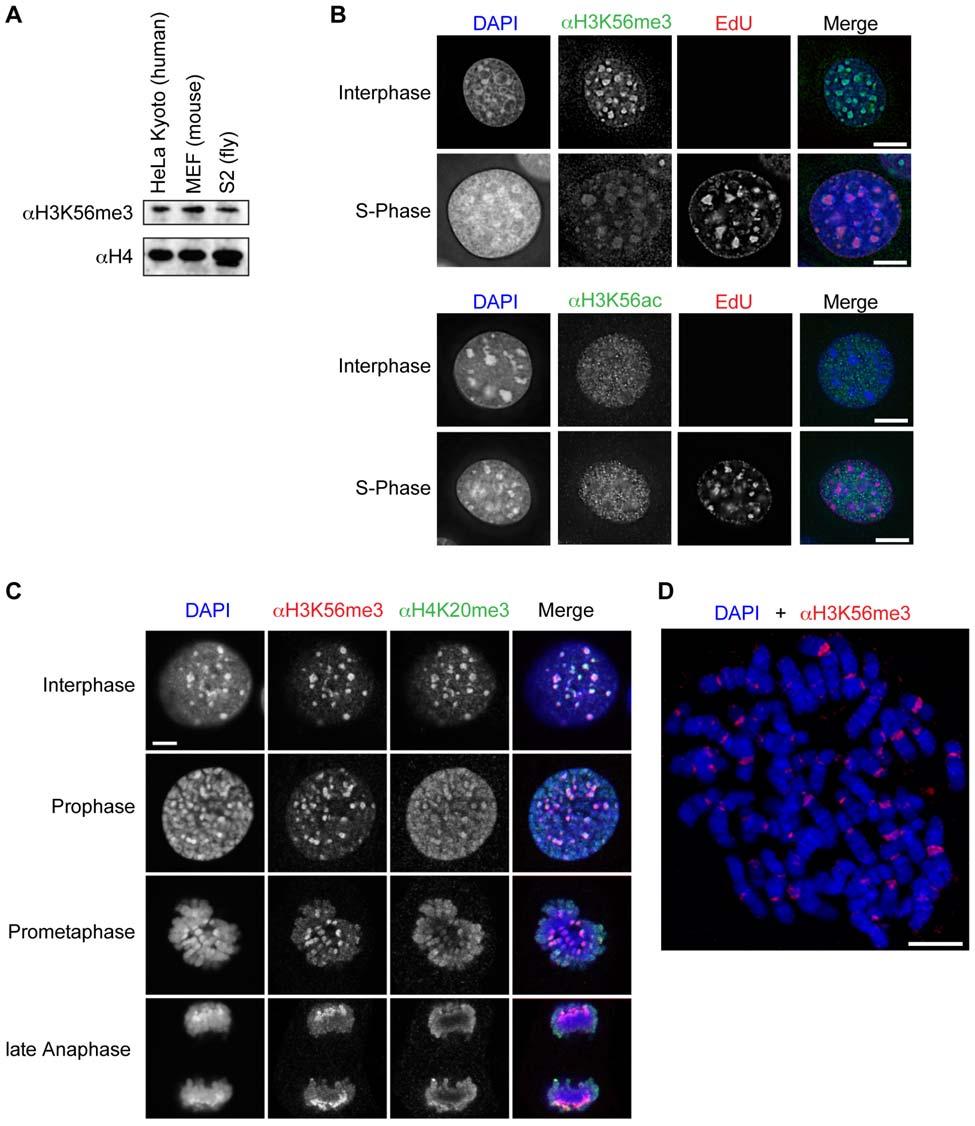
H3K56me3 is evolutionary conserved, has a cell-cycle independent appearance and is part of pericentromeric heterochromatin.

Having demonstrated the high specificity of αH3K56me3, we first examined the evolutionary occurrence of this novel mark by isolating histones from cell lines of diverse origins. Immunoblotting revealed that H3K56me3 was present in human, mouse and fly (Figure 2A), suggesting that this modification is conserved within, at least, metazoans.

Given that H3K56ac is highly conserved and that methylation and acetylation of the same residue are mutually exclusive we wanted to investigate if there were correlations between the appearance of one mark and disappearance of the other. While in yeast H3K56ac has been shown to be cell cycle dependent, showing a significant increase during S-phase, [6], [9], [25], its cell cycle distribution in mammals remains controversial [26]–[28], with a high possibility of its occurrence in all cell cycle phases [29]. Therefore, we analyzed cell cycle appearance and nuclear localization of both acetylation and methylation of H3K56 in mammalian cells. To distinguish S-phase from interphase, mouse C127 cells were pulse-labeled with the thymidine analog EdU, which was chemically coupled to a fluorescent dye using a “click-chemistry” approach [30]. Co-staining of EdU-labeled cells with αH3K56me3 revealed that, during interphase, H3K56me3 is found predominantly at DAPI-dense heterochromatic chromocenters and shows strongly diminished signal intensity in S-phase cells (Figure 2B top). Although, we observed a more or less equal appearance of H3K56ac signal in interphase and S-phase cells (Figure 2B, bottom), it is clearly distinct from the H3K56me3 signal. We also found H3K56me3 to be present throughout mitosis (Figure 2C), where it co-localizes with heterochromatin foci, in an even more precise manner than the constitutive heterochromatin marker H4K20me3 [31]. To determine H3K56me3 localization in greater detail, human metaphase chromosomes were analyzed in IF microscopy. In accordance with H3K56me3 presence at chromocenters in interphase and heterochromatin foci in mitotic cells, this modification was present in a non-random manner and found predominantly at pericentromeric heterochromatin regions that include major satellite repeats (Figure 2D). Interestingly, H3K56me3 is, in contrast to H3K9me3, rarely found at telomeres [32], suggesting that the functional roles of these two modifications in heterochromatic regions might be different.

### Mammalian methyltransferase Suv39h affects trimethylation of H3K56

**Figure 3:**
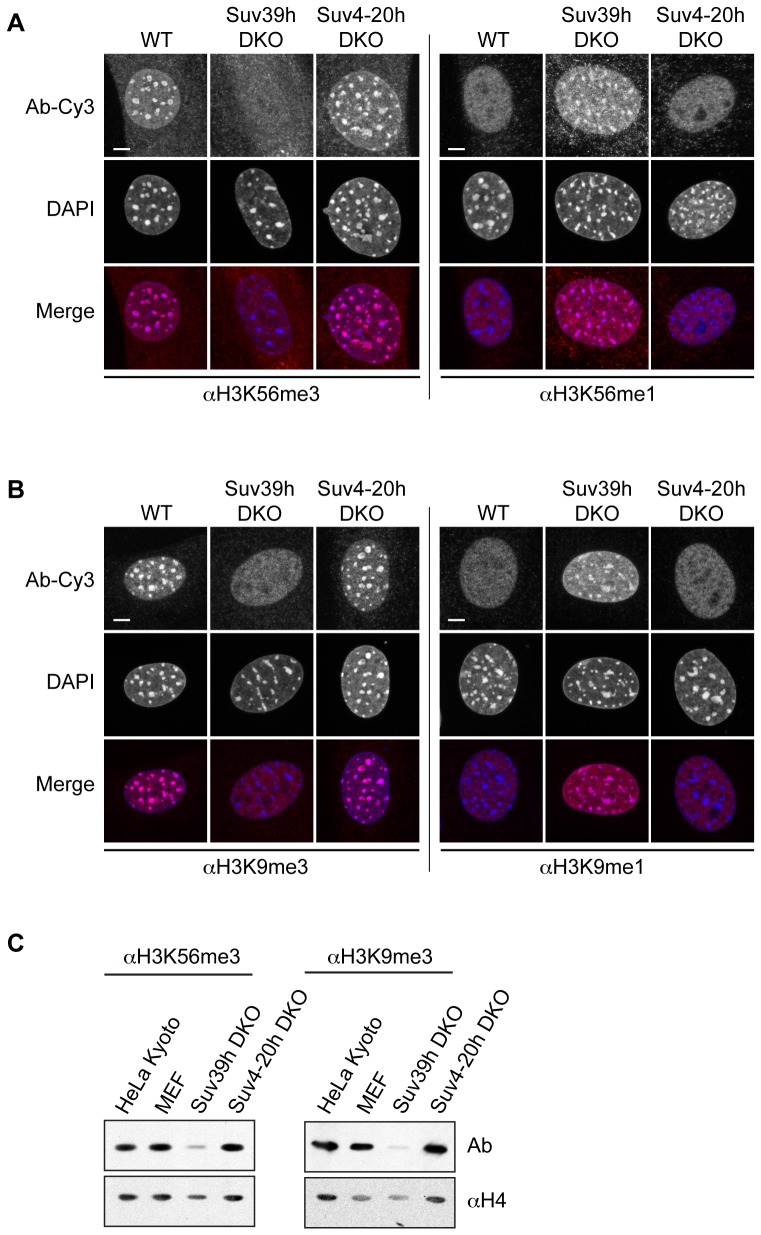
Loss of Suv39h enzymes affect H3K56me3. IF microscopy of wild type (WT), Suv39h double-null (Suv39h DKO) and Suv4-20h double-null (Suv4-20h DKO) MEF cells using various H3K56 (A) and H3K9 (B) methyl-specific antibodies (Ab-Cy3, red) and DAPI (DNA, blue). Scale bar  = 5 µm. (C) Immunoblots using acid extracted histones from HeLa Kyoto (positive control), wild type MEF, Suv39h DKO and Suv4-20h DKO cells. Blots were incubated with αH3K56me3 (left, top) or αH3K9me3 (right, top) antibodies, respectively. Blots shown at the bottom were incubated with αH4 to ensure equal loading.

To assess the functional relevance of posttranslational histone modifications, it is important to know their responsible enzymes. Several lysine methyltransferases (KMTs) that catalyze the methylation of histone lysine residues have been identified previously [33], [34]. Possibly due to the fact that both regions surrounding H3K56 and H3K9 contain a conserved K/S/T motif, monomethylation of H3K56 has been shown to be catalyzed by the H3K9me1-specific KMT G9a [12]. Additionally, both H3K9me3 and H3K56me3 localize to similar, albeit not identical, nuclear domains suggesting that H3K9 and H3K56 might share the same KMTs responsible for their trimethylation. Therefore, we first tested the H3K9me3-specific KMTs Suv39h1/2 (KMT1A and B) [35] for their ability to affect the methylation status of H3K56. Interestingly, we observed a complete loss of both H3K56me3 and H3K9me3 signals at chromocenters in Suv39h double-null MEF cells (Suv39h DKO, [14]). Accompanied with this loss of trimethyl signals, we observed an increase of the respective monomethyl marks at chromocenters (Figure 3A and B). This dramatic change in PTM localization upon the simultaneous lack of Suv39h1/2 suggests that these enzyme are involved in catalyzing trimethylation of both H3K9 and H3K56, the latter in either a direct or indirect manner. Since H3K56me3 showed a somewhat similar nuclear appearance as H4K20me3 (Figure 2C), we wondered whether Suv4-20h1/h2 enzymes, responsible for methylating lysine 20 on histone H4 [15], [31], might also target H3K56. Suv4-20h double null MEF cells (Suv4-20h DKO, [15]) showed no difference in abundance or localization of H3K56 methylation when compared to wild type cells (Figure 3A and B), demonstrating that these enzymes do not influence H3K56 methylation status. Similar results were also obtained with immunoblots, showing that the H3K56me3 signal is diminished in Suv39h DKO, but not Suv4-20 DKO acid extracted histones (Figure 3C).

### Jmjd2E/KDM4DL is a novel lysine-demethylase specific for H3K9 and H3K56 trimethylation

**Figure 4:**
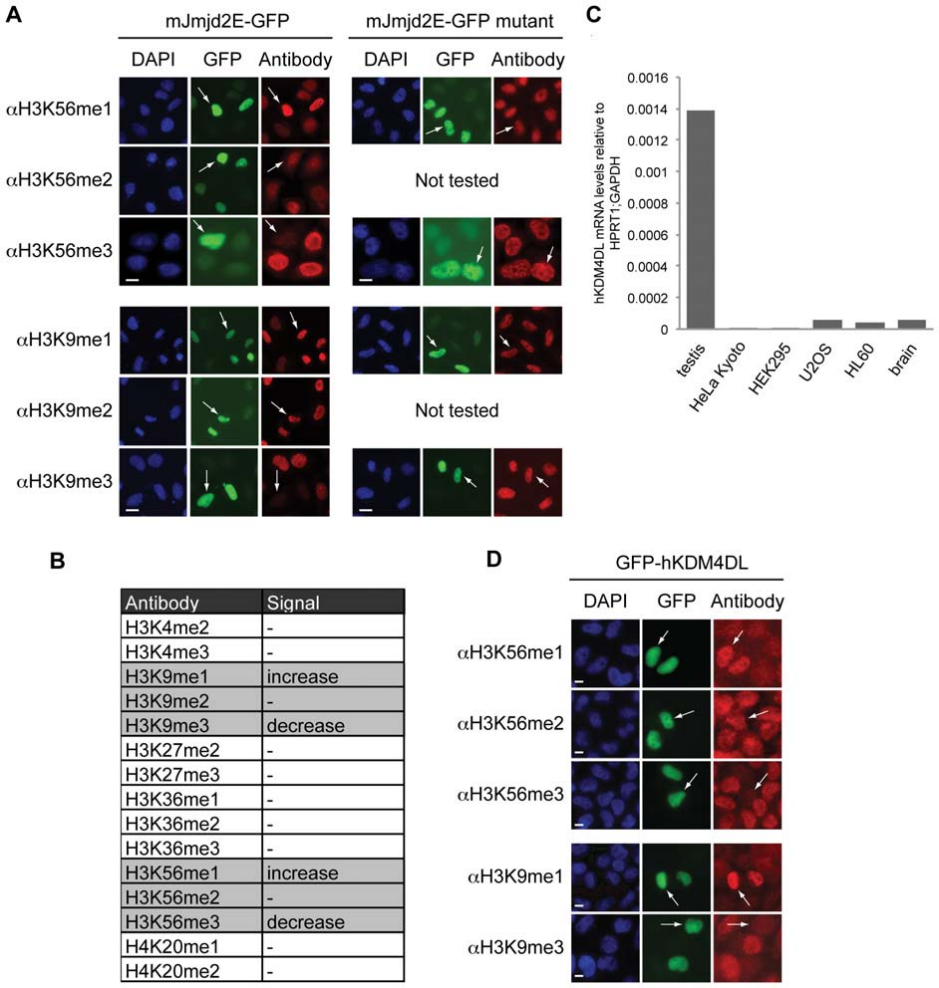
Jmjd2E demethylase affects H3K56me3. (A) IF microscopy of HeLa Kyoto cells transfected with mJmjd2E-GFP (green, left) or jmjc-domain mutated mJmjd2E-GFP (mutant, green, right) and stained with various H3K56 and H3K9 PTM-specific antibodies (red) and DAPI (DNA, blue). Arrows indicate transfected GFP-positive cells. Scale bar  = 10 µm. See also Figure S2A for IF results of cells transfected with other GFP-tagged mJMJD2 family members (mJmjd2a-d). (B) List of PTMs analyzed in IF after expression of mJmjd2E in HeLa Kyoto cells indicating changes in fluorescence intensities. See also Figure S2B for examples of IF results summarized in this table. (C) qPCR analysis with cDNAs from different human cell lines and tissues using primer pair specific for human Jmjd2E (hKDM4DL). Data were normalized to HPRT1 and GAPDH expression levels. (D) IF microscopy of HeLa Kyoto cells transfected with human GFP-hKDM4L (green) and stained with various H3K56 and H3K9 methyl-specific antibodies (red) and DAPI (DNA, blue). Arrows indicate transfected and GFP-positive cells. Scale bar  = 10 µm.

Having shown that the same enzymes that methylate H3K9 also affect trimethylation of H3K56, we wondered whether the erasure of these modifications is catalyzed by identical lysine demethylases (KDMs) as well. Histone lysines are demethylated by two different classes of enzymes that are distinguished by their enzymatic active domains and methylation-state specificities [36]. We focused our attention on the Jumonji C-terminal domain (JmjC) family of KDMs, since they are able to remove all methyl-states, including trimethylation [37]. We therefore tested a panel of GFP-tagged members of the JMJD2 group-containing demethylases that are thought to partially work on H3K9me3 [23]. Over-expression of the respective mKDM in human cells was monitored by GFP signal in IF microscopy and effects on histone methylation were analyzed by co-staining with different histone PTM antibodies. This screen led to the identification of all members of the mJMJD2 family (mJmjd2A-E) able to affect H3K56me3 (Figure S2A and Figure 4A). Since all members have previously been shown also to act on H3K9me3 [38], our results point once again towards a possible link between these two heterochromatic marks due, to a shared sequence motif (K/S/T). As one example, over-expression of mJmjd2D or mJmjd2E [39] strongly diminished H3K9me3, as well as H3K56me3 signals, in HeLa Kyoto cells (Figure 4A, left and Figure S2A). The loss of the respective trimethyl signal was accompanied with an increase in the monomethyl, but not dimethyl state, suggesting that these enzymes remove two methyl groups in total. Since over-expression of mJmjd2D-GFP caused severe cellular defects, its role on H3K56me3 was not further investigated and we focused subsequent analyses on mJmjd2E that acted solely on H3K9 and H3K56 trimethylation and not on other histone trimethylation marks (Figure 4B and Figure S2B). The observed changes in H3K9 and H3K56 methylation states upon mJmjd2E-GFP over-expression were dependent on the enzymatic active jmjC domain, since point mutations in that region completely abolished mJmjd2E's demethylase activity (Figure 4A, right). In the mouse, mJmjd2E is predicted to constitute a pseudogene and we therefore decided to analyze expression and function of the yet uncharacterized human homolog hKDM4DL. hKDM4DL mRNA is expressed predominantly in testis, with only residual levels present in U2OS (osteosarcoma) and HL60 (promyelocytic leukemia) cell lines and human brain tissue (Figure 4C). Over-expression of GFP-hKDM4DL in HeLa Kyoto cells showed identical results as seen for the mouse homolog, loss of H3K56 and H3K9 trimethylation with an accompanied gain of the respective monomethylation mark (Figure 4D). Taken together, we have identified the JMJD2 family to facilitate demethylation of H3K9 and H3K56 trimethyl states. Additionally, we showed that mJmjd2E, and its previously uncharacterized human homolog hKDM4DL, specifically remove two methyl groups from trimethylated H3K56 or H3K9 residues, depending on their catalytically active jmjC domain.

### H3K56me3 is a novel chromatin mark in *C. elegans*

**Figure 5:**
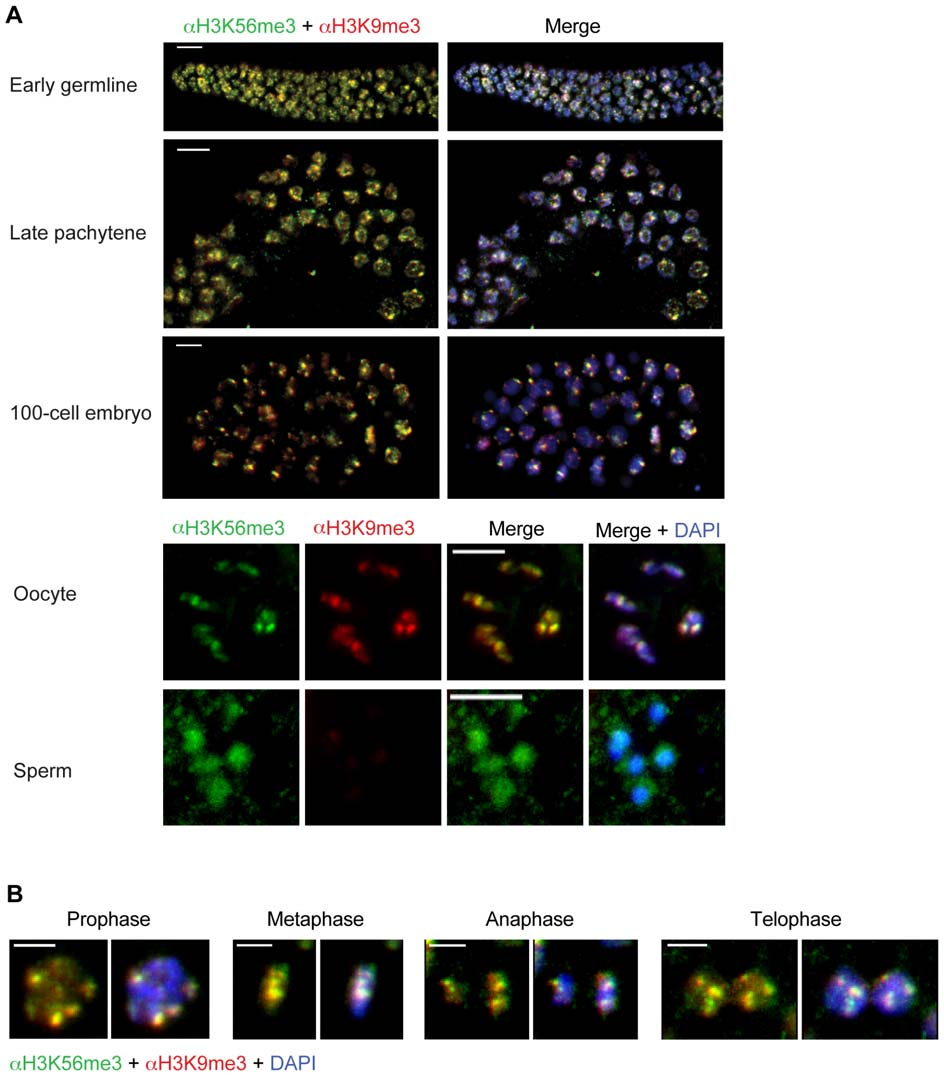
H3K56me3 is conserved in *Caenorhabditis elegans*. Shown are representative IF microscopy pictures from adult *C. elegans* hermaphrodite tissues. In all images H3K56me3 is shown in green, H3K9me3 in red, and DAPI (DNA) in blue. Scale bar  = 5 µm. A) H3K56me3 co-localizes with H3K9me3 in the early germline, late pachytene and in a 100-cell embryo (top picture). Interestingly, although H3K56me3 and H3K9me3 are both present in oocytes, only H3K56me3, but not H3K9me3, staining could be observed in sperm. (bottom, split channels) (B) H3K56me3 and H3K9me3 co-localize throughout all stages of mitosis.

In order to learn more about H3K56me3 evolutionary conservation as a novel heterochromatic histone modification and its functions, we conducted IF microscopy analysis of wild type (WT) *C. elegans* hermaphrodite germlines and embryos (Figure 5A). H3K56me3 is present in both early germline and embryonic nuclei, as marked by DAPI morphology (Figure 5A, right). In almost all cells analyzed, we observed an H3K56me3 signal that strongly co-localized with H3K9me3 in most tissues (Figure 5A). Surprisingly, H3K56me3 staining was present in both types of germline cells, oocytes and sperm, whereas the H3K9me3 signal was restricted to oocytes only (Figure 5A, bottom). These data mirror previously obtained H3K9me3 results [40] and suggest that H3K56me3 might have an important H3K9me3-independent function in sperm development. Next, we wondered whether, similar to mammalian cells, H3K56 is trimethylated in cells during mitosis in *C. elegans*. Indeed, H3K56me3 is part of all mitotic stages and overlaps with H3K9me3 signals (Figure 5B), demonstrating the evolutionary high conservation of this novel mark.

**Figure 6:**
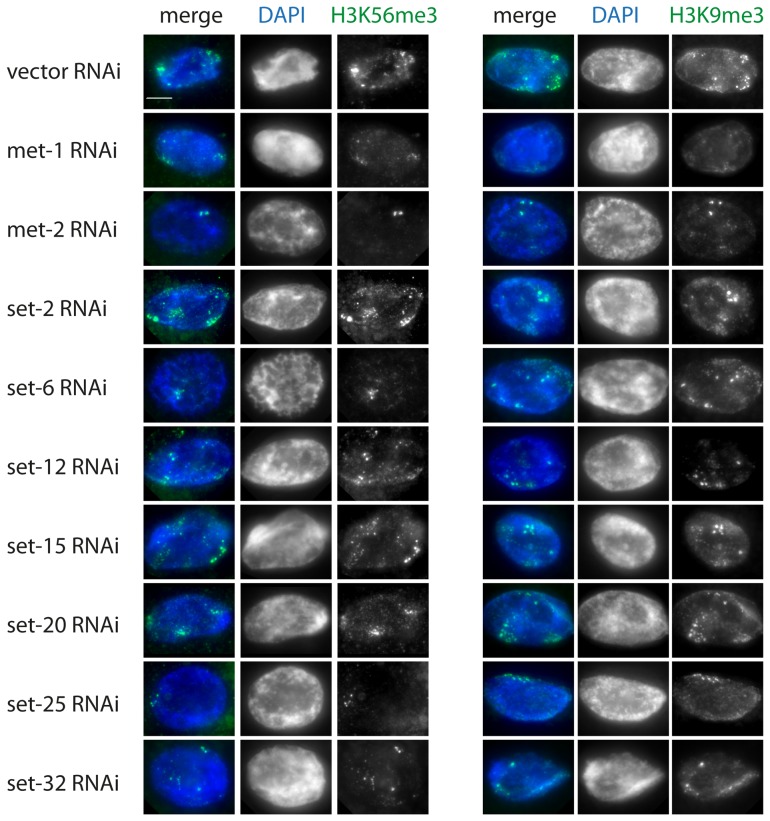
*C. elegans* RNAi screen to identify H3K56me3-specific KMTs. Shown are representative IF images from adult *C. elegans* hermaphrodite somatic intestinal nuclei following RNAi treatment. H3K56me3 (left) or H3K9me3 (right) staining is shown in green and DAPI (DNA) is shown in blue. CAPG-1 co-staining was used as a staining control (data not shown). Results show that *met-1* and *met-2* depletion severely affect both H3K56me3 and H3K9me3, while reduction of additional KMTs (*set-6, set-25* and *set-32*) has a stronger effect on H3K56me3 levels compared to H3K9me3. Scale bar  = 5 µm.

Next, we sought to shed light on the enzymatic regulation of H3K56 trimethylation in *C. elegans* and performed an RNAi-based survey of known or predicted methyltransferases, including H3K9-specific enzymes [41]. The screen included RNAi targeting MET-2, a homolog of mammalian euchromatic H3K9 HMT SETDB1 [40]–[42], MET-1, a homolog of yeast Set2, an H3K36-specific methyltransferase, whose activity was reported to be required for normal levels of H3K9me3 [41], and SET-25, a distant homolog G9a, recently reported to deposit H3K9me3 in *C. elegans* embryos [42]. We also included RNAi against previously uncharacterized SET domain containing proteins predicted to encode divergent H3K9-specific methyltransferases (set-6, -12, -15, -20, and -32). For control, we performed RNAi targeting an H3K4-specific methyltransferase SET-2, a homolog of SET1/MML. We conducted our screen in the intestine, where the large size of nuclei makes scoring easier. This screen identified several genes whose activity is required for normal levels of H3K56me3 and/or H3K9me3, some of which have been previously implicated in H3K9 methylation. H3K56me3 levels were severely reduced in *met-2* and *set-25* RNAi, consistent with the requirement for these genes for H3K9me3 levels in *C. elegans* embryos [42]. Interestingly, H3K9me3 levels were less affected in these conditions, indicating possible differences between the enzymes responsible for these marks and/or differences in antibody sensitivities. H3K56me3 levels were also reduced in *met-1* RNAi, and to a lesser extent in *set-6*and *set-32* RNAi. H3K9me3 levels were also reduced in *met-2* and *set-12* RNAi, possibly due to indirect affects (Figure 6). H3K9me3 levels were never reduced to background levels, perhaps due to partial redundancy between these enzymes. Knockdown of other known H3K9 methyltransferases or the H3K4 KMT *set-2* resulted in DAPI perturbations, but showed no effect on H3K56me3 staining (Figure 6).

In sum, H3K56me3, its relationship to H3K9me3, and its regulation by several H3K9 methyltransferases are conserved in *C. elegans*. However, some degree of divergence in the factors regulating H3K56me3 may have occurred in *C. elegans*.

## Discussion

Our study establishes the existence of a novel pericentric heterochromatin mark, H3K56me3, in several metazoan species. This novel modification is present in all cell cycle phases, with the exception of S-phase, where it is underrepresented. Enzymes targeting H3K9 also act on H3K56, as the KMTs Suv39h1/2 are important for trimethylation of both residues and KDM JMJD2 family members remove these modifications. Mouse Jmjd2E and its so far uncharacterized human homolog hKDM4DL are involved in the process of demethylating H3K56me3 to a monomethylated status. In *C. elegans*, H3K56me3 is a conserved feature of mitotic chromosomes that primarily co-localizes with H3K9me3 and is regulated by some but not all H3K9 methyltransferases.

Of particular interest is our observation in mammalian cells that H3K56me3 is found in chromocenters containing pericentric heterochromatin, but only outside of S-phase. During that particular cell cycle phase, H3K56me3-specific IF microscopy signals are strongly diminished. Such an effect can be caused either by a replication-specific removal of the trimethylation mark or by occlusion of the epitope through adjacent modifications, such as phosphorylation of H3S57, or association with a binding protein. As H3K56 is targeted by the lysine acetyltransferases CBP or GCN5 [26], [43] prior to being deposited onto DNA during replication [7], [44], [45], it is highly likely that newly synthesized H3 histones with K56ac replace “old” H3K56me3-containing ones. Given that H3K56me3 has been recently shown to prevent binding of PCNA that specifically associates with the monomethylation state [12], it is plausible that H3K56me3 needs to be removed during replication to allow proper action of PCNA at the replication forks. With regard to adjacent modification sites, a serine and a threonine, potential phosphorylation sites, are located next to lysine 56. Although H3S57 phosphorylation was reported to exist in mammals *in vivo* [46], no data on its appearance during cell cycle, on responsible enzymes and its function in mammals are available due to the lack of a specific antibody. One study, applying yeast mutants proposes a potential functional interplay between H3K56 and S57 in replicative stress recovery and transcriptional elongation [46]. However, because H3S57ph has thus far not been identified in yeast *in vivo*, it is not possible to relate such observations to the mammalian system. Concerning putative H3K56me3-specific binding partners, we applied peptide pull-down experiments followed by MS identification of precipitated proteins (data not shown). Although we repeated such experiment many times, we were not able to consistently pull-down any candidates when compared to unmodified control peptide pull-downs. It is likely that H3K56me3 is not directly recognized by any “reader” protein but, instead functions indirectly by preventing acetylation of H3K56 and its associated signaling pathways. Alternatively, since H3K56me3 is localized in the α-N-helical region near the entry-exit sites of the DNA superhelix, it is possible that the correctly folded three-dimensional structure of this region (alone or in combination with DNA or other histones) is crucial for reader binding. Therefore, the use of peptides in such pull-down experiments will not suffice in reader binding. H3K56me3 histones or even nucleosomes containing this PTM will be needed for the identification of its potential reader(s) in the future.

Our finding that H3K56me3 constitutes another heterochromatin mark is in perfect agreement with previously published data, since H3K56 is monomethylated by G9A [12] that was initially described as a KMT responsible for H3K9me1 and H3K9me2 [47]. It is therefore plausible that H3K9me3-specific KMT(s) might also act on H3K56. We report here that the loss of Suv39h enzymes leads to diminished trimethylation of both H3K56 as well as H3K9. Based on our experimental set-up using Suv39h double-null cells, it is at the moment not possible to exclude that loss of H3K56me3 stems from an indirect effect. The chance of H3K9me3 influencing trimethylation of H3K56 by an, as yet, unknown mechanism, is conceivable albeit unlikely. Several observations argue for a direct enzymatic action of Suv39h on H3K56; the presence of a “K/S/T” motif in both regions and the fact that G9a, another H3K9-specific KMT is the responsible enzyme for H3K56me1 [12]. Therefore, we propose that Suv39h enzymes directly trimethylate H3K56 leading to a pericentric heterochromatin localization.

Although like both H3K9me3 and H4K20me3, H3K56me3 also constitutes a mark found in DAPI-dense regions, these modifications are not identical in their localization when looked at in greater detail. H3K9me3 stains telomeric repeats [32] and our results indicate that the majority of H3K56me3 does not. In contrast to H4K20me3, we found H3K56me3 in distinct chromatin foci during all mitotic phases, indicating that this novel mark is found in much more distinct heterochromatic loci. We plan to investigate this finding in future studies.

Besides our discovery of a novel histone modification site, our study raises one important question for many researchers dealing with PTMs and their biological functions. The finding that some enzymes might have several targets is supported by another recent study showing that pericentric localization of H3K64me3, another H3 core modification, also depends on Suv39h activity [48]. Therefore, the observed severe knock-down [14] and over-expression [49] phenotypes that were previously assigned to the sole loss or gain of H3K9me3, respectively, have to be reevaluated, since Suv39h enzymes affect not only H3K9, but also H3K64 as well as H3K56 trimethylation, It is possible that the assigned role of H3K9me3 in protecting genome stability and heterochromatic gene silencing [50] is in part shared by H3K56me3.

In agreement with the finding that H3K9-specific KMTs act on H3K56, we demonstrated a strong correlation between both residues as to their KDM-specificity. Our study expands the list of known histone target residues of enzymes belonging to the JMJD2 family of demethylases since we could show that they act not only on H3K9me3 and, in some cases, H3K36me3 [38], but also on H3K56me3. Of particular interest is our characterization of mJmjd2E, a predicted pseudogene and its human homolog hKDM4DL, which codes for a, so far, uncharacterized protein. Because of hKDM4DL's strongest expression in human testis, it will be of great interest to determine if and why removal of the trimethylation of H3K9 and H3K56 is important in this special tissue. Perhaps it is crucial during the process of histone-protamine exchange and/or relaxation of pericentric heterochromatin in humans; a statement that will be difficult to address since the mouse enzyme is predicted to be a pseudogene and not expressed. hKDM4DL might, therefore, constitute a human or primate-specific protein. If so, then functional studies on hKDM4DL in testis will be hard, if not impossible to perform.

Our study clearly puts forward H3K56me3 as a novel modification, but we were unable to address its functional relevance. Usually, knock-down of the enzymes targeting the respective modification provide insights into its biological role; but since H3K9 and H3K56 methylations are affected by the same enzymatic machinery in mammals, we do not have any technical tool at hand to pinpoint, *in vivo*, one particular phenotype to H3K56me3. However, identification of genes that affect the two modifications slightly differently in the *C. elegans* intestine opens up the possibility of future functional studies, at least in this particular organism.

Interestingly, we identified MET-1, a H3K36 KMT homolog, as needed for wild type levels of both H3K9me3 and H3K56me3. It was previously suggested that H3K36 methylation might be a prerequisite for H3K9me3 in worms [41], and perhaps it is similarly required for H3K56me3 as well. Previous studies reported that H3K9me3 in the germline is independent of MET-2 [40], however H3K9me3 levels are significantly reduced in MET-2-depleted embryos [42]. These results indicate that different KMTs might be primarily used in different tissues. Consistent with this hypothesis, depletion of MET-2 and SET-25 significantly reduces H3K9me3 levels in embryos [42], and H3K56me3 levels in the intestine (this study), but their effect is less pronounced for H3K9me3 levels in the intestine. Future studies will be needed to reveal how the preference for different KMTs is regulated in different tissues.

We identified multiple KMTs required for normal levels of both H3K9me3 and H3K56me3. One possible explanation for the requirement of two or more methyltransferases is that one of these KMTs deposits mono- (and perhaps di-) methylation, while the second KMT deposits trimethylation, in a manner dependent on prior mono- or dimethylation. This model is similar to what was previously reported for MET-2 and SET-25 in embryos [42]. Alternative possibilities include indirect effects, perhaps involving non-histone targets for these proteins.

Early EM studies revealed that *C. elegans* embryos lack electron-dense material, classically associated with heterochromatin [51]. In addition, while in mammalian cells H3K9me3 co-localizes with DAPI-bright regions of pericentric heterochromatin, in *C. elegans*, H3K9me3 localizes to DAPI-faint regions [40], leading to the suggestion that *C. elegans* lacks heterochromatin or that heterochromatin is different in this species [40]. *C. elegans* chromosomes are holocentric, and in the absence of a localized centromere, the phrase “pericentric “ does not apply. Instead, the brightest foci of H3K9me3 in *C. elegans* nuclei associate with the nuclear lamina [42]. H3K9me3 is coincident with H3K27me3 and nuclear lamina protein LEM-1, all of which are enriched along chromosome arms [42], [52]. Therefore, these regions most likely are similar to mammalian heterochromatin near the nuclear periphery, or lamin associated domains, LADs [53]. Our results show that H3K56me3 colocalizes with H3K9me3 in worms, suggesting that H3K56me3 likely marks these lamin associated domains.

In agreement with a specialized role of H3K56me3 in testis is the finding that sperm cells in *C. elegans* contain solely trimethylation of H3K56 but not of H3K9. It will be of interest to see if H3K56me3 has an evolutionary conserved role in germline development, although its functional implication might be different in different metazoans.

## Supporting Information

Figure S1 **Immunoblot peptide competition experiments to determine specificity of αH3K9me3 antibodies used in this study.** αH3K9me3 antibodies from (A) Active Motif or (B) the Jenuwein laboratory [16] were pre-incubated with 2 µg/ml competitor peptides before addition to immunoblots containing recombinant H3 protein (R) or acid extracted HeLa Kyoto histones (H) (top). Ponceau staining (bottom) serves as loading control.
(TIF)

Figure S2 **Members of the JMJD2 family of demethylases affect H3K56me3.** (A) IF microscopy of HeLa Kyoto cells that were transfected with GFP-tagged mJmjd2a-d and human Jmjd2d homolog hKDM4 (green) and co-stained with αH3K56me3 antibody (red) and DAPI (DNA, blue). Arrows indicate transfected and GFP-positive cells. Scale bar  = 10 µm. See also Figure 4A for detailed PTM analysis of HeLa cells transfected with mJmjd2E-GFP. (B) IF microscopy of HeLa Kyoto cells that were transfected with mJmjd2E-GFP (green) and co-stained with various histone PTM-specific antibodies (red) and DAPI (DNA, blue). Arrows indicate transfected and GFP-positive cells. Scale bar  = 10 µm. See also Figure 4B that contains a listing of the results depicted here.
(TIF)

Table S1 **List of peptides used in peptide competition experiments.**
(DOCX)
