# Supplementary figures and images for: H3K56me3 Is a Novel, Conserved Heterochromatic Mark That Largely but Not Completely Overlaps with H3K9me3 in Both Regulation and Localization
Source: PLoS One. 2013 Feb 22;8(2):e51765. doi: 10.1371/journal.pone.0051765 (PMC3579866; doi:10.1371/journal.pone.0051765)

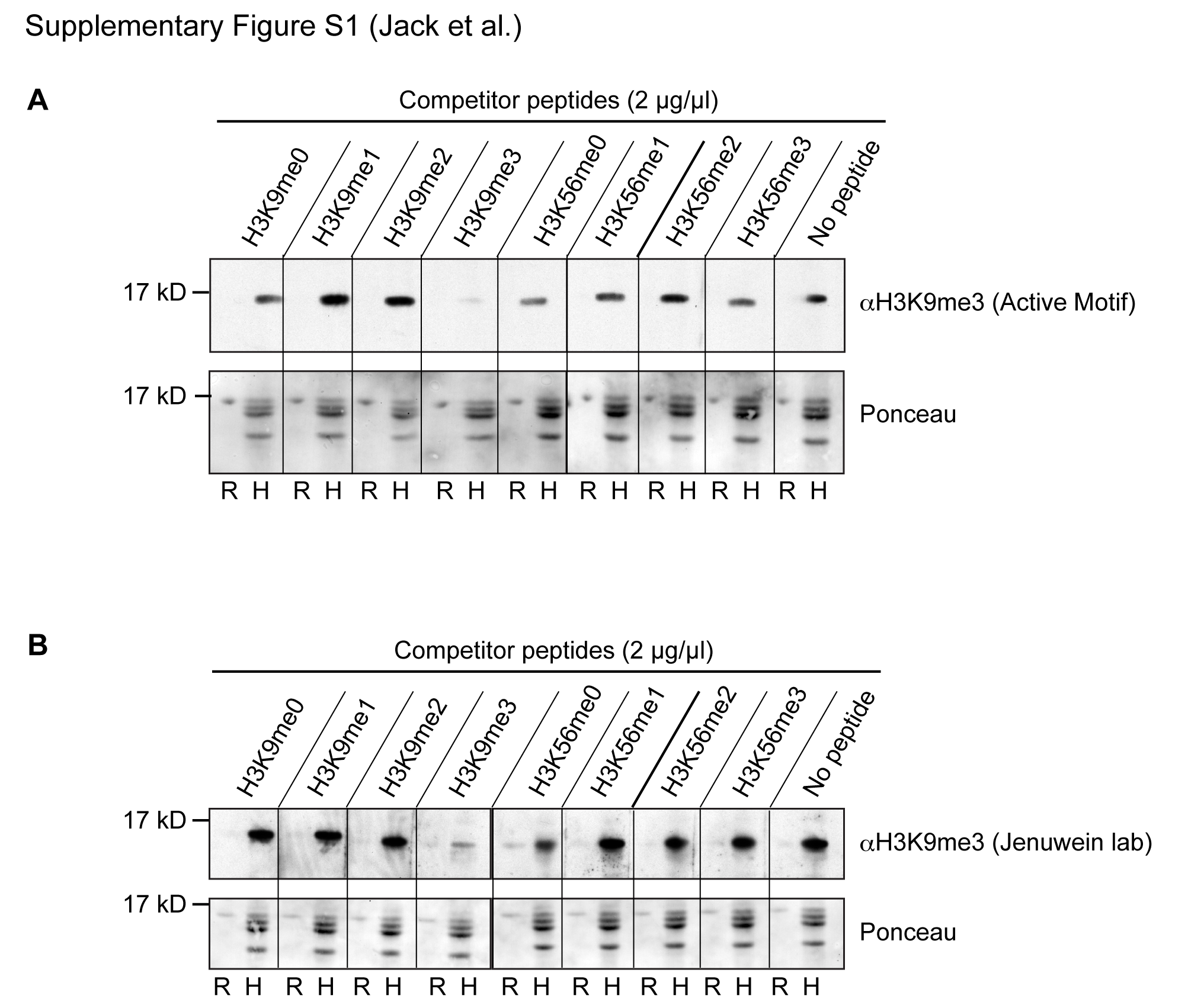

Supplement: Figure S1 — Immunoblot peptide competition experiments to determine specificity of αH3K9me3 antibodies used in this study. αH3K9me3 antibodies from (A) Active Motif or (B) the Jenuwein laboratory [16] were pre-incubated with 2 µg/ml competitor peptides before addition to immunoblots containing recombinant H3 protein (R) or acid extracted HeLa Kyoto histones (H) (top). Ponceau staining (bottom) serves as loading control. (TIF) [file pone.0051765.s001.tif]

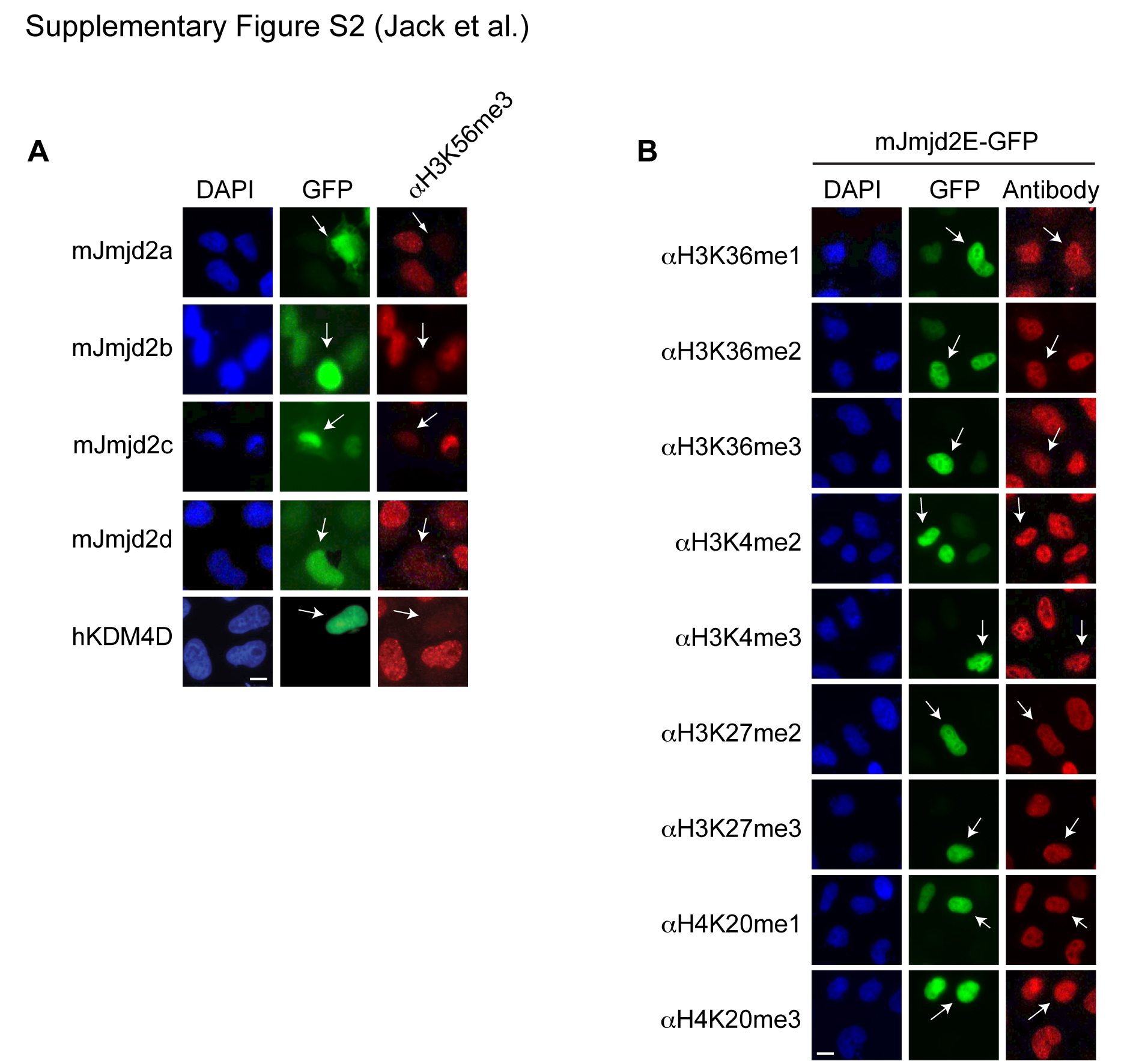

Supplement: Figure S2 — Members of the JMJD2 family of demethylases affect H3K56me3. (A) IF microscopy of HeLa Kyoto cells that were transfected with GFP-tagged mJmjd2a-d and human Jmjd2d homolog hKDM4 (green) and co-stained with αH3K56me3 antibody (red) and DAPI (DNA, blue). Arrows indicate transfected and GFP-positive cells. Scale bar = 10 µm. See also Figure 4A for detailed PTM analysis of HeLa cells transfected with mJmjd2E-GFP. (B) IF microscopy of HeLa Kyoto cells that were transfected with mJmjd2E-GFP (green) and co-stained with various histone PTM-specific antibodies (red) and DAPI (DNA, blue). Arrows indicate transfected and GFP-positive cells. Scale bar = 10 µm. See also Figure 4B that contains a listing of the results depicted here. (TIF) [file pone.0051765.s002.tif]
