# Supplementary material for: H3K56me3 Is a Novel, Conserved Heterochromatic Mark That Largely but Not Completely Overlaps with H3K9me3 in Both Regulation and Localization
Source: PLoS One. 2013 Feb 22;8(2):e51765. doi: 10.1371/journal.pone.0051765 (PMC3579866; doi:10.1371/journal.pone.0051765)
Supplement: Table S1 — List of peptides used in peptide competition experiments. (DOCX) [file pone.0051765.s003.docx]

**Supplementary Table 1 (Jack et al.)**

| Peptide | Sequence |
| --- | --- |
| H3K56me0 | VALREIRRYQKSTELLIRKL |
| H3K56me1 | VALREIRRYQK(me1)STELLIRKL |
| H3K56me2 | VALREIRRYQK(me2)STELLIRKL |
| H3K56me3 | VALREIRRYQK(me3)STELLIRKL |
| H3K9me0 | ARTKQTARKSTGGKAPRKQL |
| H3K9me1 | ARTKQTARK(me1)STGGKAPRKQL |
| H3K9me2 | ARTKQTARK(me2)STGGKAPRKQL |
| H3K9me3 | ARTKQTARK(me3)STGGKAPRKQL |
| H3K4me3 | ARTK(me3)QTARKSTGGKAPRKQL |
| H3K64me3 | YQKSTELLIRK(me3)LPFQRLVRE |
| H3K79me3 | LVREIAQDFK(me3)TDLRFQS |
